# Supplementary material for: Learning curve and surgical outcome of robotic assisted colorectal surgery with ERAS program
Source: Sci Rep. 2022 Nov 29;12:20566. doi: 10.1038/s41598-022-24665-w (PMC9709162; doi:10.1038/s41598-022-24665-w)
Supplement: Supplementary file 1 — Supplementary Information 1. [file 41598_2022_24665_MOESM1_ESM.zip › LSC Raw data-Ñ[▒K/138/N_ñw╝╨░O▒Kñσ.pdf]

W92 / W92

臺中榮民總醫院
手術室護理記錄

[redacted]

基本資料

- 室別： 二醫手術室-32室，手術科別： CRS，手術類別： 常規，日期： 2021/09/23
- 進入時間： 1200 ； 麻醉開始時間： 1200 ； Time Out時間： 1250 ； 劃刀時間： 1251 ； 手術結束時間： 1740 送出時間： 1820 ；
- 麻醉方式： GE

  診斷： Malignant tumor of rectum

  手術名稱： 1.Low anterior resection with colo-anal anastomosis2.Ileostomy3.Laparoscopy

  手術局部用藥： NIL

  術前預防性抗生素：
  - 抗生素名稱一： Veterin 1GM IVD/AR
  - 時間： 1245
  - 上次簽章: 謝孟君

  ，追加預防性抗生素：
  - 抗生素名稱一： Veterin 1GM IVD/AR
  - 時間： 1700
  - 上次簽章: 羅湳軒
- 手術醫師： 邱騰逸，蔣鋒帆，黃瑋，曾禹康
- 麻醉醫師： VS 賴慧卿，麻醉醫師： R 梁婷瑋
-
  - 刷手護士A ： 詹佳柔，時間： 1200，刷手護士B ： 王姿貽，時間： 1200，接替刷手一： 梁凱雯，時間： 1300，接替刷手二： 林采儀，時間： 1500
  - 流動護士A ： 吳明柔，時間： 1200，接替流動一： 謝孟君，時間： 1230，接替流動二： 阮鈺珊，時間： 1500
- 更多接替及流動護士： 接替刷手三： 詹佳柔，時間： 1600，接替刷手四： 阮鈺珊，時間： 1730，接替流動三： 羅湳軒，時間： 1700，接替流動四： 詹佳柔，時間： 1800

皮膚狀況

A-V Shunt： 無

術前： 完整

記錄者： 上次簽章: 謝孟君

術後： 完整

記錄者： 詹佳柔

手術準備

手術臥式 單一手術臥式

-
  - 手術臥式： 截石臥式
  - 使用附件： 溫毯 38 ℃，頭圈，腳架
  - 皮膚準備： 10%Aq Betadine，Hibiscrub，2% Chlorhexidine
  - 電燒： 使用電燒: 電極片位置： 右大腿，記錄者： 上次簽章: 謝孟君

止血帶T： 無

植入物

- 植入物名稱/號碼/數量/部位：surgiwrap/0.02*100*130mm/壹片/pelvic

，記錄者：

標本送檢

- 細菌培養 無
- 細胞學 無
- 冰凍切片 無
- 病理標本

  IMA Root

組織名稱一：

,

組織名稱二： Distal Cut End

,

組織名稱三： Rectum

- 截肢 無

標本交付 標本： ? 袋 ，標本： 零 盒 ，標本： 零 瓶 ，細胞學： 零 管 ，Culture Tube： 零 管 ，被交付醫師： R 黃瑋 ，

- 記錄者： 上次簽章: 羅渝軒
- 時間： 1750

傷口術後敷料

傷口敷料： 傷口敷料： 無

- 請於圖上標示部位：

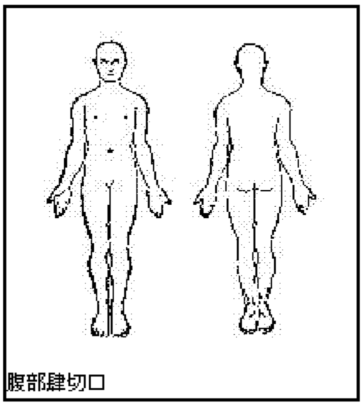

- 傷口敷料： Mepore

導管

導管	天數	導管資訊	插入日期	插入時間	更換日期	拔除日期	拔除時間	拔除原因	預計拔除/更換	預計拔除/更換日期
Hemo Vac (1)	1	號碼:19 Fr.; 部位:骨盆腔;	2021/09/23	1720						
Foley(1)	1	號碼:16 Fr.; 部位:膀胱;	2021/09/23	1225						
A-line(1)	1	號碼:20 G; 部位:左手;	2021/09/23	1215						
Rod,Stoma Supporting (1)	1	號碼:壹 ; 部位:迴腸;	2021/09/23	1700						

用物計數

- 手術中出血量： 250 c.c.
- 手術中尿量： 450 c.c.
-
    - 其它： 無

用物計數： 多切口同時縫合

- 1.器械： 正確 ，2.縫針： 正確 ，3.刀片： 正確 ，4.紗布： 正確 ，5.腹部墊： 未使用 ，6.紗球： 未使用 ，7.PEANUTS ： 未使用 ，8.紗條： 未使用 ，9.絲帶： 未使用 ，10.針頭： 未使用 ，11.Vessel loop： 未使用 ，12.Boots： 未使用 ，13.Jelco： 未使用 ，14.棉條： 未使用 ，
    - 第一次：
        - 刷手護士 ： 詹佳柔
        - 流動護士： 上次簽章: 羅渝軒
        - 時間： 1700

    ,
    - 第二次：
        - 刷手護士 ： 詹佳柔
        - 流動護士： 上次簽章: 羅渝軒
        - 時間： 1720

    ,
    - 第三次：

- 刷手護士 ： 阮鈺珊
- 流動護士： 上次簽章: 羅渝軒
- 時間： 1735

備註

備註： NIL

- 記錄者： 上次簽章: 羅渝軒
- 時間： 1755

填寫護理人員 詹佳柔

正式摘要

W92 / W92

doctorRank:B
